# Coexistence of condyloma acuminatum and extramammary Paget’s disease on penis and scrotum: A rare case report

**DOI:** 10.1097/MD.0000000000031754

**Published:** 2022-11-11

**Authors:** Inho Kang, Joon Ho Lee, Jong Im Lee, Joon Shik Hong, Young Woong Mo, Gyu Yong Jung

**Affiliations:** a Department of Plastic and Reconstructive Surgery, College of Medicine, Dongguk University, Gyeongju-si, Republic of Korea; b Pathology, College of Medicine, Dongguk University, Gyeongju-si, Republic of Korea; c Department of Plastic and Reconstructive Surgery, Institute for Human Tissue Restoration, Severance Hospital, Yonsei University College of Medicine, Seoul, Republic of Korea.

**Keywords:** case report, condyloma acuminatum, extramammary Paget disease, human papillomavirus

## Abstract

Extramammary Paget’s disease (EMPD) is a rare skin cancer that commonly occurs in sites rich in apocrine glands, such as perineum, vulva, axilla, scrotum, and penis. On the other hand, condyloma acuminatum (CA; also referred to as anogenital warts) is a common benign neoplasm caused by human papillomavirus. Few cases of coexistent EMPD and CA have been reported because of the rarity of the condition.

**Patient concerns and diagnosis:**

A 72-year-old man with a genital mass, which appeared to be composed of multiple papillomatous masses, was referred for surgical resection. The lesion was first noticed 6 months previously and grew rapidly. CO_2_ ablative laser therapy was performed twice at a primary clinic, but the mass recurred.

**Intervention and outcomes:**

Excisional biopsy revealed the presence of coexistent EMPD and CA. Additional wide excision was performed, and postoperative biopsy confirmed no residual tumor. Two years after surgery, no recurrence had occurred.

**Lessons:**

CA can co-occur with several types of skin malignancies, and a skin malignancy coexisting with CA is difficult to diagnose visually. Therefore, even if a skin lesion in the genital region is considered to be CA, we recommend punch biopsy before treatment because it can benefit prognosiss.

## 1. Introduction

Extramammary Paget’s disease (EMPD) is a rare skin cancer that arises by epidermotrophic spread from an in situ or invasive neoplasm arising in an adnexal gland within dermis.^[1]^ Therefore, the sites most commonly affected by EMPD are rich in apocrine glands, such as perineum, vulva, axilla, scrotum, and penis.^[1–3]^ The most common presenting symptom of EMPD is pruritis, and clinically it is usually visualized as a demarcated, thickened, eczematoid, or crusted lesion with an irregular border.^[1]^ Lesions are occasionally hyper- or hypo-pigmented.^[1]^ Furthermore, EMPD is difficult to distinguish visually from other skin lesions such as eczema, contact dermatitis, or Bowen’s disease,^[1,4]^ and therefore, its diagnosis is often delayed. Pathologically, neoplastic cells exhibiting glandular differentiation are observed in the form of intraepithelial (usually intraepidermal) infiltrations,^[1]^ and thus cytokeratin 7, which is expressed in sebaceous glands and the secretory coils of eccrine glands, is a specific immunohistochemical marker for EMPD.^[5]^

Condyloma acuminata (CA; anogenital warts) is usually caused by human papillomavirus strains 6 or 11, though uncommonly by strains 16, 18, 31, 33, or 35.^[6]^ CA is usually asymptomatic, though this depends on lesion size and anatomical location, but can be pruritic or painful.^[6–9]^ Clinically, CA is usually encountered as a papillomatous eruption in genital mucosa and is usually diagnosed by visual inspection. Only rarely is its diagnosis confirmed by biopsy. Currently available treatments for CA focus on wart removal rather than eradicating the underlying viral infection.^[8]^ CA is treated using topical agents (podophyllotoxin, imiquimod cream, or sinecatechins), by destructive or surgical treatments (trichloroacetic acid, cryotherapy, CO_2_ laser ablation, or surgery), or by systemic interferon treatment.^[6–9]^

Only 3 cases of coexistent CA and EMPD have been reported.^[10–12]^ Here, we report a rare case of coexistent EMPD and CA.

## 2. Case presentation

A 72-year-old Asian male patient with hypertension visited our urology department with multiple papillomatous masses of penis and scrotum. The patient reported he first noticed the lesion 6 months previously and that it later increased considerably in size. Initially, he visited a primary clinic where the masses were diagnosed as CA by visual inspection. CO_2_ ablative laser therapy was performed twice, but the mass recurred. Accordingly, he was referred to our institute for excision and reconstruction.

**Figure 1.**
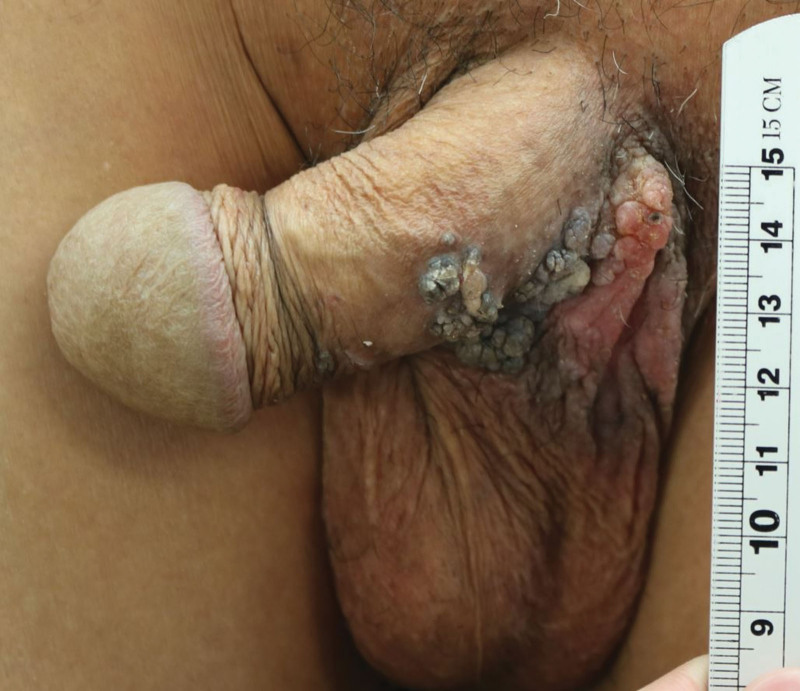
Preoperative photograph. 8.0 × 6.0 cm lesion on penis and scrotum with hypopigmented plaque and multiple papillomatous masses.

**Figure 2.**
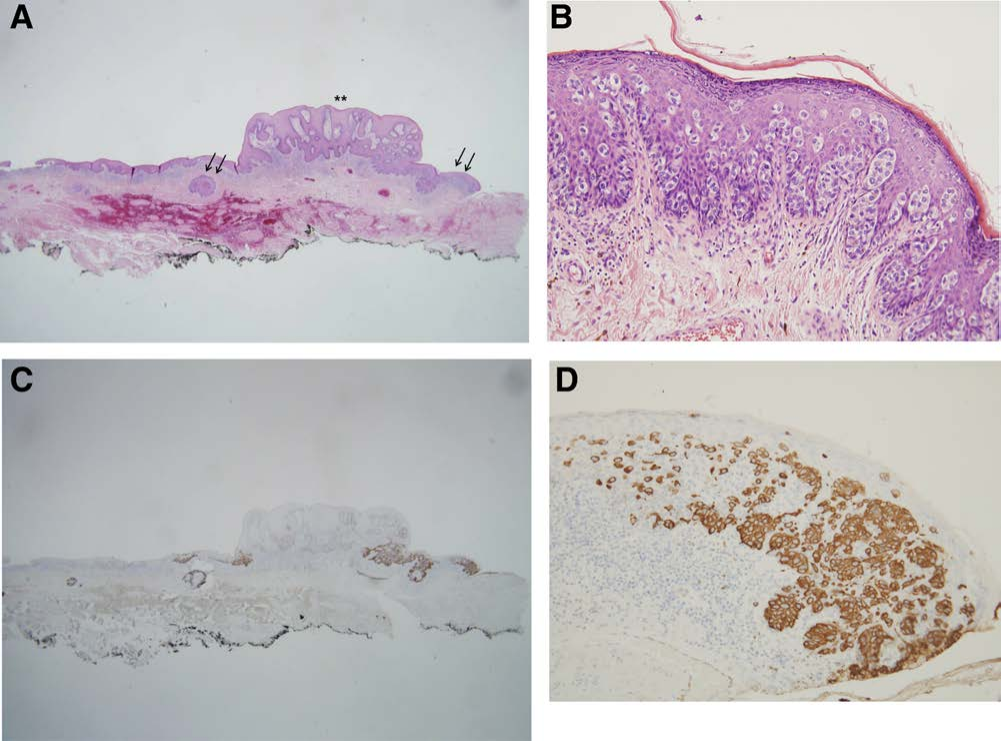
Histologic features. (A) A polypoid lesion of condyloma acuminatum was noted (asterisks), and the adjacent surface and follicular epithelium were thickened (arrows) (H&E, ×40). (B) At high magnification, large, atypical cells with abundant cytoplasm (Paget cells) were observed in thickened epithelium (H&E, ×200). (C, D) Immunohistochemical staining for allowed Paget cells, which presented as single cells or nests, to be easily identified at the periphery and base of the condyloma and adjacent thickened epithelium (CK7, ×40/CK7, ×200). CK7 = cytokeratin 7.

**Figure 3.**
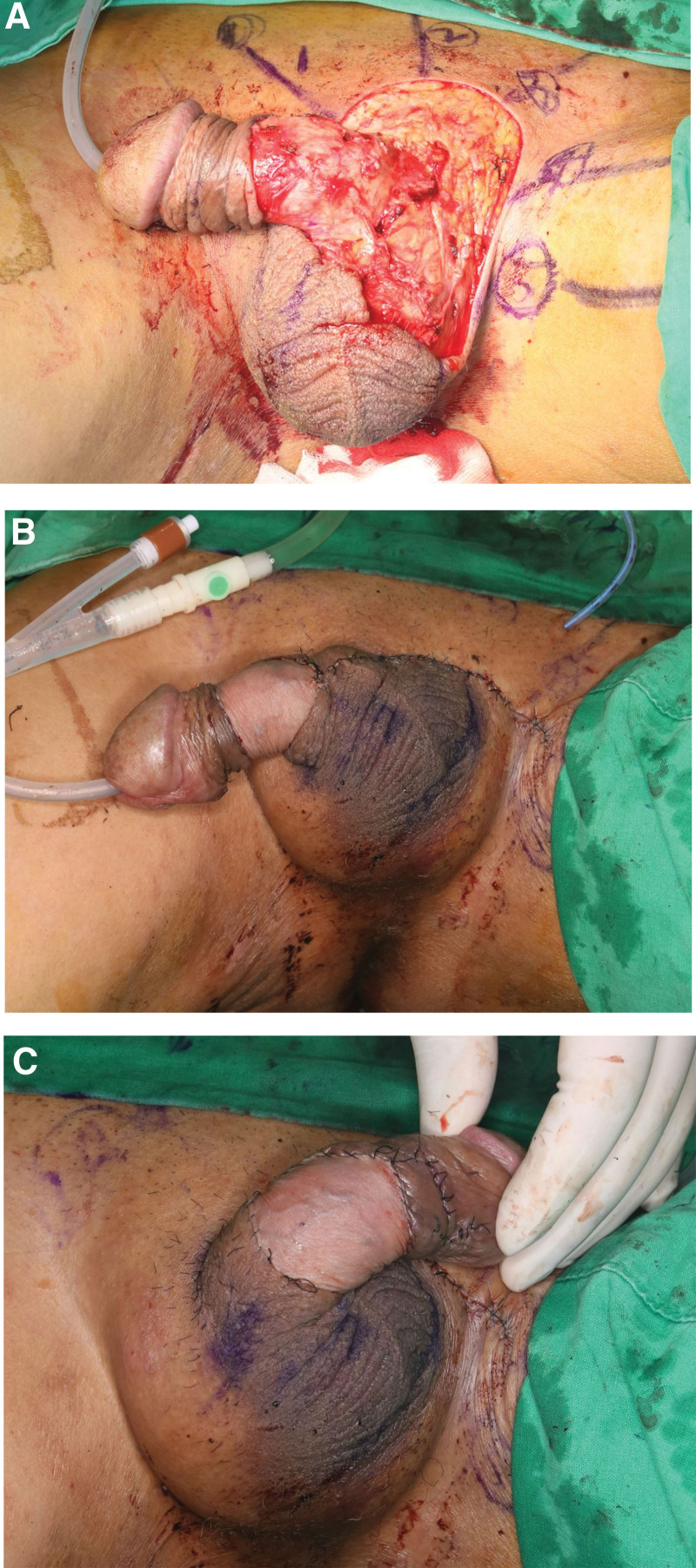
Intraoperative photographs. (A) Wide excision was performed with a margin of 2 cm. Excision was performed until the entire margin was negative, as determined by frozen biopsy. (B, C) Defects in the scrotum and abdomen were covered using a scrotal dartos musculocutaneous flap, and the penile shaft was covered using a full-thickness skin graft.

**Figure 4.**
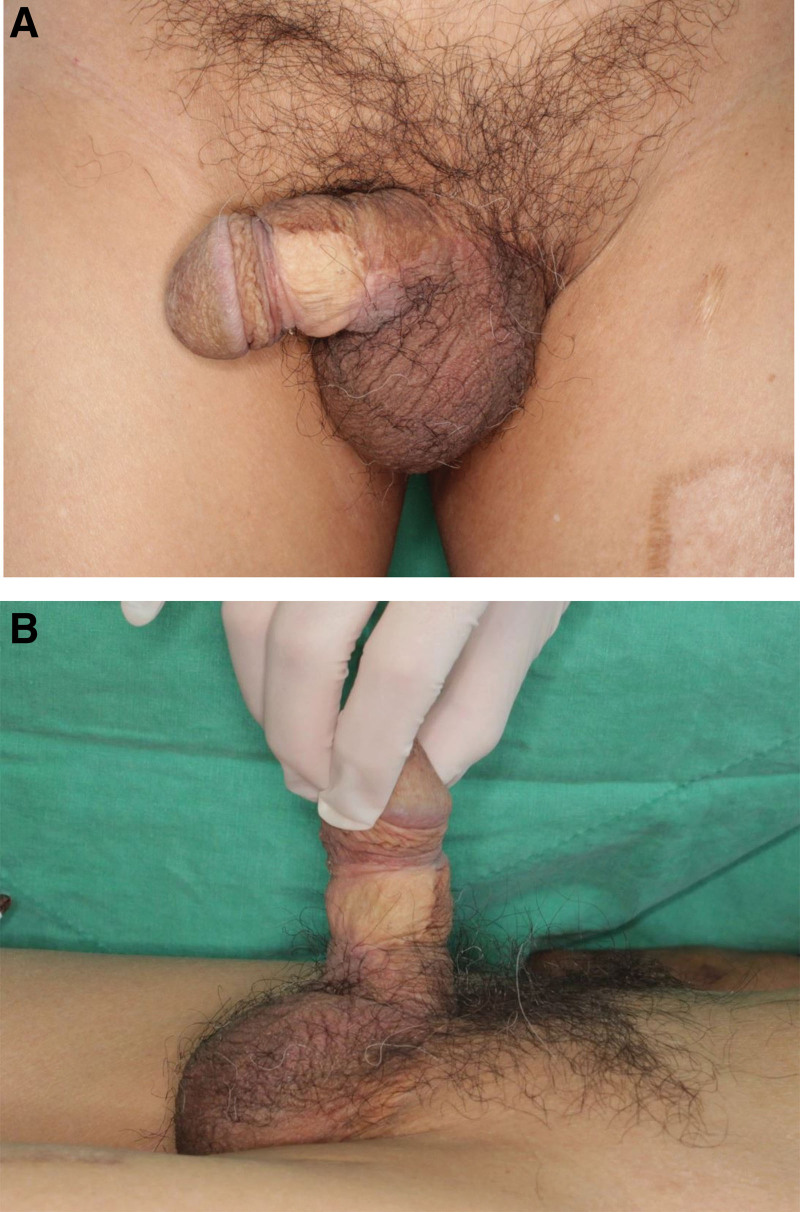
Postoperative photographs. (A) No recurrence occurred during 2 yr of postoperative follow-up, and no wound complication was encountered. (B) FTSG reconstruction of the penile shaft provided sufficient elasticity to preserve erection function. FTSG = full-thickness skin graft.

Physical inspection revealed a hypopigmented eczematoid plaque lesion (approximately 8 × 6 cm in size) and a verrucous papule above the lesion (Fig. 1). Excision of the lesion and defect coverage with a local flap was planned, and surgery was performed under spinal anesthesia. However, postoperative biopsy results revealed the presence of coexistent CA and EMPD (Fig. 2), the presence of residual tumor in all margins, and that the tumor was confined to epidermis. Chest and abdominopelvic computed tomography were performed to exclude possible distant metastasis, but none was observed. An oncologist consulted regarding the need for adjuvant therapy recommended that additional lymph node dissection and adjuvant therapy were not required and that treatment could be terminated after complete resection. We decided to excise remaining EMPD with a margin of 2 cm, and excision was performed until frozen biopsy showed that margins were negative for EMPD (Fig. 3A). In addition, defects in scrotum and abdomen were covered using a scrotal dartos musculocutaneous flap, and the penile shaft was covered using a full-thickness skin graft (Figs. 3B and C). No recurrence was evident 2 years after surgery (Fig. 4A), and no wound complication was encountered. Furthermore, full-thickness skin graft reconstruction of the penile shaft provided sufficient elasticity to preserve erection function (Fig. 4B).

## 3. Discussion

Only 3 cases of coexistent condyloma acuminatum and EMPD have been previously reported.^[10–12]^

EMPD is a slowly insidiously progressive intraepithelial skin cancer, and if left untreated and distant metastasis occurs prognosis is poor. EMPD is difficult to distinguish from other skin lesions visually and should be diagnosed by biopsy.^[1,3]^ Various therapeutic modalities have been suggested, including wide excision, laser ablation, radiotherapy, chemotherapy, and topical agent application,^[1,3,13]^ but in noninvasive EMPD cases, wide local surgical excision with an adequate margin (2–3 cm) is the treatment of choice.^[3,13]^ If distant metastasis is suspected, sentinel lymph node biopsy and lymph node dissection may be performed,^[13]^ and depending on the situation, radiotherapy or chemotherapy may be used. Noninvasive EMPD can be cured by wide local excision alone, but the prognosis of invasive EMPD is dismal. According to a recent study, the 5-year mortality rate of EMPD exhibiting deep dermal invasion was 85.71%.^[14]^ Therefore, early detection and treatment are critical.

CA can be treated with topical agents, laser ablation, cryotherapy, or surgery, although most primary clinics initially adopt a method less invasive than surgery. Biopsies are rarely performed. However, though less invasive methods destroy CA and surrounding tissues, they may mask a coexistent skin malignancy. Therefore, in cases of coexistent CA and skin cancer (EMPD, Bowen’s disease, basal cell carcinoma, or squamous cell carcinoma), a diagnosis of skin cancer is likely to be delayed. Many reports of Bowen’s disease, basal cell carcinoma, or squamous cell carcinoma accompanying CA have been issued.^[2,11,15]^ The Centers for Disease Control and Prevention guidelines for anogenital warts recommend that biopsy be performed when a lesion is atypical (e.g., pigmented, indurated, affixed to underlying tissue, bleeding, or ulcerated).^[6]^ When skin cancer is missed, diagnosis is inevitably made in a more advanced state. Furthermore, visual diagnosis of a skin malignancy coexisting with CA without a biopsy is difficult, and thus, we recommend a biopsy be conducted before treating condyloma non-surgically.

In previous case reports, authors have discussed the possibility that CA and EMPD may interact synergistically during disease progression.^[10–12]^ In our case, a pathologic examination showed CA and EMPD lesions were clearly demarcated, which suggested it was unlikely that CA influenced the occurrence of EMPD, but as EMPD weakens nearby cell immunity, human papillomavirus infection may have initiated CA.^[10,16]^

## 4. Conclusion

CA can co-occur with several types of skin malignancies, but it is difficult to diagnose a skin malignancy coexisting with CA visually. Therefore, even if a skin lesion in the genital region is considered to be CA, we recommend punch biopsy before treatment because it can substantially benefit prognosis.

## Author contributions

**Conceptualization:** Inho Kang.

**Data curation:** Inho Kang, Jong Im Lee, Joon Ho Lee.

**Formal analysis:** Inho Kang.

**Supervision:** Joon Ho Lee, Gyu Yong Jung.

**Writing – original draft:** Inho Kang.

**Writing – review & editing:** Inho Kang, Joon Ho Lee, Jong Im Lee, Young Woong Mo, Joon Shik Hong, Gyu Yong Jung.
